# Estimating quality adjusted progression free survival of first-line treatments for EGFR mutation positive non small cell lung cancer patients in The Netherlands

**DOI:** 10.1186/1477-7525-10-108

**Published:** 2012-09-10

**Authors:** S Cora Verduyn, Bonne Biesma, Franz MNH Schramel, Feike W van der Scheer, Merel K Langenfeld, Maria A de Peuter, Anne-Marie C Dingemans

**Affiliations:** 1 Mapi Consultancy, Houten, The Netherlands; 2 Department of Pulmonology, Jeroen Bosch Hospital, 's-Hertogenbosch, The Netherlands; 3 Department of Pulmonology, Sint Antonius Hospital, Nieuwegein, The Netherlands; 4 AstraZeneca NL, Zoetermeer, The Netherlands; 5 Department of Pulmonology, Maastricht University Medical Center, Maastricht, The Netherlands

**Keywords:** Advanced non-small cell lung cancer, Tyrosine kinase inhibitors, EGFR mutation, Gefitinib, Quality of life, Progression free survival

## Abstract

**Background:**

Gefitinib, a tyrosine kinase inhibitor, is an effective treatment in advanced non-small cell lung cancer (NSCLC) patients with an activating mutation in the epidermal growth factor receptor (EGFR). Randomised clinical trials showed a benefit in progression free survival for gefitinib versus doublet chemotherapy regimens in patients with an activated EGFR mutation (EGFR M+). From a patient perspective, progression free survival is important, but so is health-related quality of life. Therefore, this analysis evaluates the Quality Adjusted progression free survival of gefitinib versus three relevant doublet chemotherapies (gemcitabine/cisplatin (Gem/Cis); pemetrexed/cisplatin (Pem/Cis); paclitaxel/carboplatin (Pac/Carb)) in a Dutch health care setting in patients with EGFR M+ stage IIIB/IV NSCLC. This study uses progression free survival rather than overall survival for its time frame in order to better compare the treatments and to account for the influence that subsequent treatment lines would have on overall survival analysis.

**Methods:**

Mean progression free survival for Pac/Carb was obtained by extrapolating the median progression free survival as reported in the Iressa-Pan-Asia Study (IPASS). Data from a network meta-analysis was used to estimate the mean progression free survival for therapies of interest relative to Pac/Carb. Adjustment for health-related quality of life was done by incorporating utilities for the Dutch population, obtained by converting FACT-L data (from IPASS) to utility values and multiplying these with the mean progression free survival for each treatment arm to determine the Quality Adjusted progression free survival. Probabilistic sensitivity analysis was carried out to determine 95% credibility intervals.

**Results:**

The Quality Adjusted progression free survival (PFS) (mean, (95% credibility interval)) was 5.2 months (4.5; 5.8) for Gem/Cis, 5.3 months (4.6; 6.1) for Pem/Cis; 4.9 months (4.4; 5.5) for Pac/Carb and 8.3 (7.0; 9.9) for gefitinib.

**Conclusions:**

In the Dutch health care setting, the previously established progression free survival benefit of first-line gefitinib in advanced NSCLC EGFR M+ patients in comparison to standard doublet chemotherapy is further supported by the Quality Adjusted PFS, which takes into account the additional health-related quality of life benefits of gefitinib over doublet chemotherapy.

## Background

Gefitinib is a selective small molecule inhibitor of the epidermal growth factor receptor (EGFR) tyrosine kinase (TK); it is an effective treatment for patients with advanced non small cell lung cancer (NSCLC, stage IIIb/IV, new TNM classification stage IV [1]) and activating mutations of the EGFR TK [2-5].

The European Medicines Agency (EMA) approval for gefitinib treatment in advanced NSCLC in patients with EGFR mutation-positive (M+) tumours was based largely on evidence from the Iressa Pan-Asia Study (IPASS) [5], together with a comprehensive review of gefitinib data in EGFR M+ NSCLC patients across lines of therapy. In IPASS, a combination of paclitaxel and carboplatin (Pac/Carb) was compared to gefitinib for first-line treatment of clinically-selected advanced NSCLC patients [5]. The pre-planned subgroup analysis of the EGFR M+ patients in this study demonstrated that gefitinib had a significantly longer progression free survival (PFS) period than Pac/Carb (HR 0.48 (95% Confidence Interval: 0.36; 0.64), median PFS 9.5 months and 6.3 months, respectively [5,6]). Gefitinib was also associated with a lower rate of common terminology criteria (CTC) for grade 3 and 4 adverse events (AE)[5]. The PFS results and lower incidence of AE for gefitinib were confirmed by other trials in EGFR M+ patients, which compared either Pac/Carb [3] or other standard doublet chemotherapies such as gemcitabine/cisplatin (Gem/Cis) [7] or cisplatin/docetaxel [4] to gefitinib. The increase in median PFS with gefitinib ranged from 1.8 [7] to 4.9 months [3].

Though the significant benefit in PFS was clear, there did not appear to be a similar overall survival (OS) benefit of gefitinib over doublet chemotherapy [3,4,7,8]. In the IPASS, the median OS was 21.6 months for gefitinib and 21.9 for Pac/Carb (p = 0.990)[8]. One reason for the similar OS could be that all studies allowed for further treatments at disease progression, including a cross-over where patients on chemotherapy could cross-over to gefitinib or another tyrosine kinase inhibitor (TKi) and vice versa [3-5,7,8]. Second line therapy will affect OS; this makes it difficult to interpret OS differences between initial treatments. Therefore, in this situation, PFS may be considered a more appropriate measure of the true effect of first-line treatment.

When considering treatment effect from a patient perspective, not only is the length of (progression free) survival important; health-related quality-of-life (HRQoL) during that period is also important. HRQoL was measured with disease-specific HRQoL instruments in two studies; IPASS used the FACT-L and First-SIGNAL used the EORTC QLQ-C30 and QLQ-LC13. Both studies demonstrated an improved HRQoL with gefitinib treatment over doublet chemotherapies [5,7,9].

It is important for a new drug to show added value in comparison to standard care. QALYs are a recognised and established measure of disease burden, including both quantity-of-life (mean life-years) and quality-of-life, and are therefore a useful means of expressing the value of a new therapy. One measure of quality of life is through the evaluation of utilities. Utility is a measure of preference, and ranges from 0 (death) to 1 (full health).

How, then, should gefitinib be evaluated in comparison to standard care? Standard first-line care for advanced NSCLC in the Netherlands is Gem/Cis or pemetrexed/cisplatin (Pem/Cis) doublet chemotherapy [10]. The CEGEDIM 2008 also reported first-line off label prescription of TKi’s for advanced NSCLC [10]. While there is a lack of utility data for both gefitinib and standard first-line doublet chemotherapies for advanced NSCLC, the FACT-L data from the IPASS study can be transformed into utility data using a published and widely recognised algorithm [11]. Furthermore, a recent Dutch study by Grutters et al. evaluated the utilities among survivors of NSCLC [12] (predominantly stage I, II and IIIa) and found that HRQoL in NSCLC patients is influenced by the occurrence of adverse events and objective response.

The objective of this study is to evaluate the Quality Adjusted PFS of gefitinib versus relevant doublet chemotherapies in the Netherlands in patients with EGFR M+ stage IIIb/IV NSCLC during the progression free state.

## Methods

When demonstrating the added value of a treatment, a relevant time period should be used that covers all costs and benefits for that disease. In oncology, a life time horizon is often applied. However, in the NSCLC studies used for this analysis, OS may be largely influenced by the effect of subsequent treatment lines introduced at disease progression. Therefore, this analysis measures the true effect of gefitinib as a first line therapy by exploring preferences/utilities in a Dutch treatment context during the progression free time frame.

### Calculation of mean PFS for first-line therapy

In order to calculate the mean PFS for first-line therapy, this analysis uses data from the gefitinib single technology appraisal (STA) submission to the National Institute for Health and Clinical Excellence (NICE) in the UK. For this STA, a cost-effectiveness model was developed to compare gefitinib to other doublet chemotherapies in first-line treatment of EGFR M+ advanced NSCLC patients [6]. To support this submission, in the absence of a head-to-head trial, a network meta-analysis (NMA) for all standard doublet chemotherapies for stage IIIb/IV NSCLC relative to Pac/Carb [6] was performed to establish the relative efficacy and safety of treatments. This NMA was based on a systematic literature search performed in May 2009. In the same STA submission, a meta-analysis was performed to estimate the relative effects of gefitinib to Pac/Carb in EGFR M+ patients, using data from the IPASS study [5] and the North East Japan Study group trial [3,6]. The studies reported by Mitsudomi et al. [4] and Lee et al. [7] were not used, since they did not use Pac/Carb, but other doublet chemotherapies. The NMA assumed that the relative effect of chemotherapies is not influenced by EGFR mutation status. Table 1 summarises the HRs for PFS and odds ratios for objective response derived in the UK NMA for all treatments of interest [6].

**Table 1:** HR, and odds ratios obtained with the NMA for duration of PFS and OR, all relative to Pac/Carb treatment

	**Pac/Carb**	Gem/Cis*	Pem/Cis*	Gefitinib**
PFS HR (95% CrI)	1	0.92 (0.80; 1.04)	0.88 (0.74; 1.05)	0.43 (0.34; 0.53)
OR (Odds ratio) (95% CrI)	1	1.16 (0.93; 1.44)	1.64 (1.15; 2.27)	4.63 (3.01; 6.98)

HR:Hazard ratio, PFS: progression free survival, OR: objective response Data retrieved from UK NMA [6] 95% CrI: 95% credibility interval, * determined for general patient group with advanced NSCL and first-line treatment, ** determined for EGFR M+ patients.

For an economic evaluation, the median PFS was translated into a mean PFS. In order to calculate the mean PFS for all treatments of interest, the HR from Table 1 was applied to an estimated mean PFS for the baseline therapy (Pac/Carb). The mean PFS for Pac/Carb was obtained by extrapolating the median PFS as reported in the IPASS study using a Weibull regression model. In the Technical Support document 14 of DSU about survival analysis [13], the Weibull distribution is the most commonly used distribution within submissions to NICE. The modelled Weibull curve showed a good fit with the Kaplan Meier PFS curve (almost complete overlap).

### Calculation of utility data for the Netherlands

When estimating the preference for a certain health state in the Netherlands, preferences provided by the general Dutch public are needed. Hence, to assess the utilities for Dutch advanced NSCLC EGFR M+ patients, 11 items of the FACT-L questionnaire data from the subgroup of EGFR M+ patients (n = 261) in the progression free period in the IPASS study were converted into Dutch utilities by applying the unequal distribution algorithm published by Lamers et al. [11].

FACT-L data for each EGFR M+ patient from the IPASS study were obtained for both arms at randomisation, at 1 week of treatment, and then at 3, 6, 9, 12, 15, 18, 24, 30, 36 and 42 weeks, up until progression of disease. For each patient, both the utility at each time point and the change from baseline (CFB) were obtained. The mean difference and standard deviation were calculated and time points were weighted by the number of participants available at each time point. To calculate statistical significance between utility CFB, an unpaired t-test was used.

In the absence of data for all comparators, the utility value calculated for Pac/Carb in the progression free period in IPASS was also used for the other doublet chemotherapies (Pem/Cis and Gem/Cis). This might provide an underestimation of utility values for the other doublet chemotherapies; the implications of this assumption are explored in the discussion.

### Calculation of quality adjusted life years in PFS

To estimate the QALYs for the progression free period, the treatment arm-specific utilities were multiplied with the estimated mean PFS to calculate the Quality Adjusted PFS for each treatment.

Since no other utility data for the Netherlands exists we have compared our findings with the utilities used in the model presented in the NICE STA submission [6]. These utilities are based on British preference weights. The estimation of the utility during PFS from the NICE STA consists of four components: stable baseline disease (i.e. state of disease without any change in pre-treatment condition), objective response, effect of drug administration, and effect of AE. Patients with stable disease have a utility of 0.653 [14]; objective response gives an increment of 0.053 (mean of Nafees et al. [14] and Doyle et al. [15]). The mode of administration of the drug also influenced utility, with a decrement of 0.043 for intravenous therapy and 0.014 for oral therapy [16].

Adverse events lead to a decrement ranging from 0.03 for rash to 0.09 for neutropenia [14]; all adverse events were set to occur in the first cycle.

## Results

### Mean PFS for first-line therapy

Table 2 presents the mean PFS as calculated with the cost effectiveness model for the three doublet chemotherapies and gefitinib. Doublet chemotherapy results were 6.7 months for Pac/Carb (95% Credibility Interval (CrI): 5.9; 7.4), 7.0 months for Gem/Cis (95% CrI: 6.1; 7.9) and 7.2 months for Pem/Cis (95% CrI: 6.2; 8.2). The mean PFS with gefitinib was 10.5 months (95% CrI: 8.9; 12.6). This was significantly higher than the doublet chemotherapies and is in line with the HR of 0.43 for gefitinib versus 0.92 for Gem/Cis and 0.88 for Pem/Cis.

**Table 2:** PFS and Quality Adjusted PFS (QA-PFS) in months (mean and 95% CrI)

	**Pac/Carb**	**Gem/Cis**	**Pem/Cis**	**Gefitinib**
PFS duration (months)	6.7 (5.9; 7.4)	7.0 (6.1; 7.9)	7.2 (6.2; 8.2)	10.5 (8.9; 12.6)
QA-PFS (months)	4.9 (4.4; 5.5)	5.2 (4.5; 5.8)	5.3 (4.6; 6.1)	8.3 (7.0; 9.9)
ΔQA-PFS with gefitinib (months)	3.4 (2.4; 4.8)	3.2 (2.0; 4.6)	3.0 (1.9; 4.4)	-

### Utility data for Netherlands

**Figure 1:**
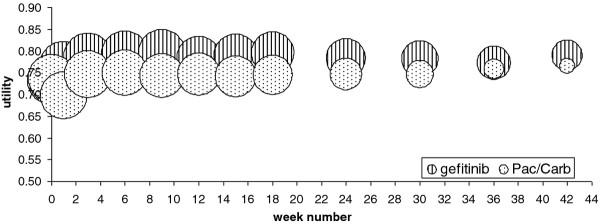
**Utility per treatment arm during progression free state in IPASS in EGFR M+ patients.** Bubble size reflects number of patients; at baseline (week number 0), both bubbles completely overlap.

In the IPASS study used for utility data in this analysis, 251 of the 261 EGFR M+ patients completed the FACT-L questionnaire at baseline, and at least once during treatment in the progression free state. The baseline utility was 0.736 ± 0.1059 for all EGFR M+ patients. At baseline, no difference was present between the two treatment arms. Figure 1 shows the utilities for both treatment arms, together with the number of patients in the progression free state at each time point (reflected by bubble size). In the gefitinib arm, utility increased after start of treatment; after 3 weeks, a steady state level was reached. The number of patients in the progression free state slowly declined from 128 at baseline and week 1 to 50 at week 42. The Pac/Carb arm showed a decline in utility in the first week; thereafter, the utility increased again and stabilised for the remainder of the progression free period. Patient numbers in the progression free state ranged from 123 at baseline and week 1 to 14 at week 42. At all time points in the progression free state, there was a significant difference between the utilities of the gefitinib and the doublet chemotherapy arms (unpaired t-test P < 0.00001). The weighted mean for the CFB in utilities associated with being treated with gefitinib was 0.0528 ± 0.0095; for Pac/Carb, this was 0.0011 ± 0.018.

### Quality adjusted PFS

In order to obtain the Quality Adjusted PFS, the specific utilities in each treatment arm were multiplied with the estimated mean PFS to calculate the Quality Adjusted PFS. The Quality Adjusted PFS was 4.9 months (95% CrI: 4.4; 5.5) for Pac/Carb, 5.2 months (95% CrI: 4.5; 5.8) for Gem/Cis, 5.3 months (95% CrI: 4.6; 6.1) for Pem/Cis, and 8.3 months (95% CrI: 7.0; 9.9) for gefitinib. This resulted in a gain of Quality Adjusted PFS ranging between 3.0 and 3.4 months for gefitinib in comparison to standard first-line treatments (doublet chemotherapies) (Table 2).

When using the UK utilities from the NICE STA submission, comparable results were obtained. However, baseline utility was lower, and therefore absolute gain in Quality Adjusted PFS with gefitinib was lower (Table 3). Regardless of which utilities were used, Quality Adjusted PFS increased with gefitinib treatment in comparison to doublet chemotherapy.

**Table 3:** Outcomes for PFS and QA PFS in an alternative scenario with UK utilities (mean number of months and 95% CrI)

	**Pac/Carb**	**Gem/Cis**	**Pem/Cis**	**Gefitinib**
PFS (months)	6.7 (6.0; 7.4)	7.0 (6.1; 7.9)	7.2 (6.2; 8.2)	10.5 (8.9; 12.4)
QALY associated with duration of PFS (months)	4.4 (3.9; 4.8)	4.6 (4.0; 5.2)	4.7 (4.0; 5.4)	6.9 (5.8; 8.1)
QALY increment associated with objective response in PFS	0.17 (0.13; 0.20)	0.19 (0.15; 0.24)	0.22 (0.17; 0.28)	0.39 (0.31; 0.49)
QALY adjustment due mode of administration	−0.16 (−0.16; -0.15)	−0.16 (−0.17; -0.15)	−0.16 (−0.17; -0.15)	−0.15 (−0.17; -0.12)
QALY decrement associated with adverse events in PFS	−0.051 (−0.055; -0.047)	−0.054 (−0.063; -0.045)	−0.060 (−0.076; -0.046)	−0.006 (−0.004; -0.007)
Total QA-PFS (months)	4.3 (3.8; 4.8)	4.5 (3.9; 5.2)	4.7 (4.0; 5.4)	7.1 (6.0; 8.4)
ΔQA-PFS with gefitinib (months)	2.8 (1.9; 3.9)	2.6 (1.6; 3.7)	2.4 (1.4; 3.6)	-

## Discussion

### Overall results

In this study, we estimated the Quality Adjusted PFS for patients with advanced NSCLC and EGFR M+ in the Netherlands. The estimated mean PFS was significantly longer with gefitinib than with standard doublet chemotherapy. Using Dutch preference weights from the FACT-L in the EGFR M+ patients in the IPASS study resulted in a baseline utility of 0.74. First-line treatment with gefitinib results in a utility increment of 0.053. The mean PFS in combination with the calculated utilities resulted in a Quality Adjusted PFS of 8.3 months for gefitinib compared to a range of 4.9 to 5.3 months for the three doublet chemotherapies considered as comparators. This is a relative gain of ≈ 50% for PFS and a relative gain of ≈ 60% for quality adjusted PFS, emphasising the better quality of life for patients treated with gefitinib compared to doublet chemotherapy. These results are in line with the significant prolongation of the time to FACT-L deterioration in the EGFR M+ patients in the gefitinib arm compared with the Pac/Carb arm [9].

### Merits and limitations of Quality Adjusted PFS

When assessing the value of a new drug in oncology, it is generally recommended to look at the consequences of that new drug treatment over a lifetime horizon. This requires inclusion of all lines of treatment in a disease and the use of OS as endpoint. However, using OS as an endpoint has some drawbacks when illustrating the value of a treatment, especially since OS is not always clearly linked to first-line treatment when multiple treatment lines can be given. The use of second and subsequent lines of treatment affects OS in both arms, particularly when there is significant cross-over to the alternative treatment. In this specific EGFR M+ patient population, relatively high response rates were seen when gefitinib was given as a second-line treatment [3]. However, the response to second-line chemotherapy after gefitinib was lower than response to second-line gefitinib after chemotherapy; in Maemondo et al., the response rate to second-line chemotherapy was 28.8%, while it was 58.8% for second-line gefitinib [3]. Because there is little second-line treatment response data available from other RCTs, we could not fully analyse gefitinib’s effect on OS while taking into account the presence and type of second-line therapy.

If there is indeed a lower response to second-line treatment for standard doublet chemotherapy than for second line gefitinib, this might explain the lack of survival benefit of first-line gefitinib versus standard doublet chemotherapy in the gefitinib studies, despite the clear benefit in PFS for gefitinib [3-5]. Support for this assumption can be found in the study by Rosell et al. [17]; erlotinib (another TKi), had a similar response rate in EGFR M+ patients when used in first-line and second-line treatment (73.5% (95% CI: 64.1; 81.2) and 67.4% (95% CI: 57.3; 76.0), respectively) [17]. Patients progressing after first-line erlotinib and receiving second-line doublet chemotherapy experienced a response rate of only 33% [17]. The reported OS for patients treated with erlotinib was similar for first and second-line treatment (28 and 27 months, respectively).

In economic evaluations, the lack of OS data for specific treatment arms requires a high amount of assumptions to be incorporated into the analysis, often by assuming that different treatment sequences of first and second line treatments result in a similar OS [18]. This assumption weakens the value of the results of the OS evaluation, since survival variables are highly influencing these results.

If in the current model next to progression free time, post progression time with the use of OS NMA [6] is also taken into account, the increase in QALY is 0.22 (95% CrI: -0.06;0.54) resulting in a cost per QALY for a lifetime horizon of 69,478€ (95% CrI: -148,134;42,191). This large credibility interval due to the high uncertainty rate makes it difficult to use this as a valid outcome.

Using PFS as a primary outcome in this field is becoming more common. A recent first-line treatment trial with bevacizumab in advanced NSCLC changed the primary outcome from OS to PFS [19]. Next to the possibility of earlier publication, one major decision point was the possible confounding of an OS endpoint by use of second-line therapy [19]. As in our analysis, when OS results were analysed, no OS benefit was found for bevacizumab despite the PFS benefit [20].

Other authors have discussed whether PFS data is acceptable to FDA and EMA (19) and have noted difficulties in reconciling positive PFS results with the lack of clear benefit in OS outcomes (20).

Clearly, in some analysis situations, using Quality Adjusted PFS during the progression free stage is a viable alternative to using QALYs and a life time horizon.

### Limitations of this study

A possible limitation of this study is the use of the Pac/Carb derived utility as a proxy for other doublet chemotherapies. In Europe, Pac/Carb is not often used and in the NMA other doublet chemotherapies showed a higher objective response. An objective response to therapy results in an increase in utility [14,15]. In Table 3, all components of the UK domains of the utility value in the PFS are presented; the differences in objective response are responsible for 0.06 months between Pac/Carb and Pem/Cis. Considering the total difference between chemotherapy and gefitinib, such difference is considered minimal.

The baseline utility value of the current study is comparable with the 0.73 utility value of Lamers et al. [11]. Baseline values reported in the UK for this patient group are lower, with Nafees at 0.65 [14] and Doyle at 0.67 [15]. There are two possible reasons for this difference. The NSCLC patient group for which Nafees et al. [14] determined preference weights received second-line treatment, i.e., they had progressed from their initial chemotherapy and were possibly feeling sicker than patients starting with first-line therapy. Another explanation could be differences in preference weights between the Dutch and British population [11].

When using country-specific preference weights to calculate QA-PFS , the differences in QA-PFS between treatment arms can differ in magnitude. This is clearly visible in Tables 2 and 3 when looking at (total) QA-PFS and ΔQA-PFS with gefitinib. The values of QA-PFS for the various treatment arms differ between the calculations for the Netherlands and the UK. For instance, (total) QA-PFS of gefitinib for the Netherlands is 8.3 (7.0; 9.9) months, whereas for the UK the QA-PFS is 7.1 (6.0; 8.4) months. Not only the absolute values of QA-PFS per treatment arm are different, but also the relative differences between treatment arms within one country are different, as can be seen for ΔQA-PFS with gefitinib for Pac/Carb: 3.4 (2.4; 4.8) months in the Dutch scenario versus 2.8 (1.9; 3.9) months for the UK scenario. Highlighting these differences emphasises the value of using country-specific utility data in health economic analyses and decision making.

Adverse events do influence utility. In NSCLC, decrements for AE vary from 0.35 for severe AE (such as dyspnoea grade ≥3)[12], to decrements of 0.03 for rash, to a range from 0.09 to 0.27 for neutropenia [14-16]. Pain, cough and dyspnoea resulted in decrements of 0.04 to 0.069 [15].

However, since AEs do not last the whole progression free period, their absolute impact is small. The different AE profiles of the three comparator chemotherapies are therefore not considered a major influence on Quality Adjusted PFS, as is also shown with the small effect of AE on outcome when we use actual AE data and the UK utilities (see Table 3). Our calculations assume that AEs are reflected in the FACT-L score; when looking at the chemotherapy arm, a utility decrement in the first week of treatment can be seen, which returned to baseline after 3 weeks. This could be due to the occurrence of AE in the first week of chemotherapy.

## Conclusions

In conclusion, the PFS benefit of first-line gefitinib in advanced Dutch NSCLC EGFR M+ patients in comparison to doublet chemotherapy is further supported by the calculation of Quality Adjusted-PFS in the Dutch health care setting, which takes account of the additional HRQoL benefits for gefitinib over doublet chemotherapy.

## Abbreviations

AE: Adverse events; CFB: Change from baseline; CTC: Common terminology criteria; EGFR: Epidermal growth factor receptor; EGFR M+: Activated EGFR mutation; EMA: European Medicines Agency; Gem/Cis: Gemcitabine/cisplatin; HR: Hazard ratio; HRQoL: Health-related quality-of-life; Pem/Cis: Pemetrexed/cisplatin; IPASS: Iressa-Pan-Asia Study; M+: Mutation-positive; NICE: National Institute for Health and Clinical Excellence; NMA: Network meta-analysis; NSCLC: Non-small cell lung cancer; OS: Overall survival; Pac/Carb: Paclitaxel/carboplatin; PFS: Progression free survival; QALYs: Quality-of-life years; STA: Single technology appraisal; TKi: Tyrosine kinase inhibitor.

## Competing interests

For the last 12 months Dr. B. Biesma declared no conflict of interest. Dr. AM. Dingemans received research funding from AstraZeneca and Roche, she served on the advisory boards of AstraZeneca, Boehringer Ingelheim, Eli Lilly, Roche, and Merck.

Dr. F. Schramel served on the advisory boards of AstraZeneca, and Eli Lilly.

F. van der Scheer and M. Langenfeld are employed by AstraZeneca Netherlands, R. de Peuter and S. Verduyn are employed by Mapi Consultancy, a consultancy which offers services to the pharmaceutical industry.

## Authors’ contributions

All authors were in involved in the conception and design of the manuscript, next to this SCV and FvdS were responsible for the acquisition, analysis and interpretation of the data, RdP and ML were involved in interpretation of the data. SCV drafted the manuscript, all other authors made substantial revisions to the manuscript and all authors have given final approval of the version to be submitted. All authors read and approved the final manuscript.

## References

[B1] D'Addario G Fruh M Reck M Baumann P Klepetko W Felip E Metastatic non-small-cell lung cancer: ESMO clinical practice guidelines for diagnosis, treatment and follow up Ann Oncol 2010 21 SUPPL 5 v116 v119 Date of Publication: May 2010 2010;v116-v119 20555059 10.1093/annonc/mdq189

[B2] Gupta A Raina V Geftinib J Cancer Res Ther 2010 6 249 254 10.4103/0973-1482.73330 21119248

[B3] Maemondo M Inoue A Kobayashi K Sugawara S Oizumi S Isobe H Gemma A Harada M Yoshizawa H Kinoshita I Fujita Y Okinaga S Hirano H Yoshimori K Harada T Ogura T Ando M Miyazawa H Tanaka T Saijo Y Hagiwara K Morita S Nukiwa T Gefitinib or chemotherapy for non-small-cell lung cancer with mutated EGFR N Engl J Med 2010 362 2380 2388 10.1056/NEJMoa0909530 20573926

[B4] Mitsudomi T Morita S Yatabe Y Negoro S Okamoto I Tsurutani J Seto T Satouchi M Tada H Hirashima T Asami K Katakami N Takada M Yoshioka H Shibata K Kudoh S Shimizu E Saito H Toyooka S Nakagawa K Fukuoka M Gefitinib versus cisplatin plus docetaxel in patients with non-small-cell lung cancer harbouring mutations of the epidermal growth factor receptor (WJTOG3405): an open label, randomised phase 3 trial Lancet Oncol 2010 11 121 128 10.1016/S1470-2045(09)70364-X 20022809

[B5] Mok TS Wu YL Thongprasert S Yang CH Chu DT Saijo N Sunpaweravong P Han B Margono B Ichinose Y Nishiwaki Y Ohe Y Yang JJ Chewaskulyong B Jiang H Duffield EL Watkins CL Armour AA Fukuoka M Gefitinib or carboplatin-paclitaxel in pulmonary adenocarcinoma N Engl J Med 2009 361 947 957 10.1056/NEJMoa0810699 19692680

[B6] AstraZeneca NICE, SINGLE TECHNOLOGY APPRAISAL (STA) for Gefitinib for the first line treatment of locally advanced or metastatic non-small lung cancer 2010 http://www.nice.org.uk/nicemedia/live/12185/47251/47251.pdf 10.3310/hta14suppl2/10 21047494

[B7] Lee JS Park K Kim SW Lee DH Kim HT Han YT Yun T Ahn JS Suh C Lee JS Yu SY Han JH Lee JW Sook JJ A randomized Phase III study of gefitinib (IressaTM) versus standard chemotherapy (Gemcitabine and Cisplatin) as a first line treatment for never smokers with advanced or metastatic adenocarcinoma of the lung 2009 San Francisco: 13th Biennial World Conference on Lung Cancer of the International Association for the Study of Lung Cancer (IASLC)

[B8] Yang C-H Fukuoka M Mok TS Wu Y-L Thongprasert S Saijo N Chu D-T Jiang H Duffield EL Ichinose Y Final overall survival (OS) results from a phase iii, randomised, open-label, first-line study of gefitinib (G) v carboplatin/paclitaxel (C/P) in clinically selected patients with advanced nonsmall cell lung cancer (NSCLC) in Asia (IPASS) Ann Oncol 2010 21 viii1 viii2

[B9] Thongprasert S Duffield E Wu Y Yang C Saijo N Chu DT Chan V Mok T Magill P Fukuoka M Quality Of Life (Qol) In A Randomized Phase III First-Line Study Of Gefitinib (G) Vs Carboplatin/Paclitaxel (CP) In Clinically Selected Asian Patients (Pts) With Advanced NSCLC (IPASS) J Thorac Oncol 2010 5 S80 S81

[B10] Praktijkonderzoek CDN Customer information 2008 Naarden, Netherlands: Cegedim 2008

[B11] Lamers LM Uyl-de Groot CA Buijt I The use of disease-specific outcome measures in cost-utility analysis: the development of Dutch societal preference weights for the FACT-L scale Pharmacoeconomics 2007 25 591 603 10.2165/00019053-200725070-00005 17610339

[B12] Grutters JP Joore MA Wiegman EM Langendijk JA de Ruysscher D Hochstenbag M Botterweck A Lambin P Pijls-Johannesma M Health-related quality of life in patients surviving non-small cell lung cancer Thorax 2010 65 903 907 10.1136/thx.2010.136390 20861294

[B13] Latimer N NICE DSU technical support document 14: survival analysis for economic evaluations alongside clinical trials - extrapolation with patient-level data http://www.nicedsu.org.uk/NICE%20DSU%20TSD%20Survival%20analysis_finalv2.pdf 27905716

[B14] Nafees B Stafford M Gavriel S Bhalla S Watkins J Health state utilities for non small cell lung cancer Health Qual Life Outcomes 2008 6 84 10.1186/1477-7525-6-84 18939982 PMC2579282

[B15] Doyle S Lloyd A Walker M Health state utility scores in advanced non-small cell lung cancer Lung Cancer 2008 62 374 380 10.1016/j.lungcan.2008.03.019 18467000

[B16] Tabberer M Stamuli E Walker M Summerhayes M Lees M Utilities associated with Non-Small Cell Lung Cancer (Nsclc): a community study Value Health 2006 9 A298

[B17] Rosell R Moran T Queralt C Porta R Cardenal F Camps C Majem M Lopez-Vivanco G Isla D Provencio M Insa A Massuti B Gonzalez-Larriba JL Paz-Ares L Bover I Garcia-Campelo R Moreno MA Catot S Rolfo C Reguart N Palmero R Sanchez JM Bastus R Mayo C Bertran-Alamillo J Molina MA Sanchez JJ Taron M Screening for epidermal growth factor receptor mutations in lung cancer N Engl J Med 2009 361 958 967 10.1056/NEJMoa0904554 19692684

[B18] de Lima LG Jr Segel JE Tan DS Do YK Mok T Finkelstein EA Cost-effectiveness of epidermal growth factor receptor mutation testing and first-line treatment with gefitinib for patients with advanced adenocarcinoma of the lung Cancer 2012 118 1032 1039 10.1002/cncr.26372 21792863

[B19] Reck M von Pawel J Zatloukal P Ramlau R Gorbounova V Hirsh V Leighl N Mezger J Archer V Moore N Manegold C Phase III trial of cisplatin plus gemcitabine with either placebo or bevacizumab as first-line therapy for nonsquamous non-small-cell lung cancer: AVAil J clin oncol 2009 27 1227 1234 10.1200/JCO.2007.14.5466 19188680

[B20] Reck M von Pawel J Zatloukal P Ramlau R Gorbounova V Hirsh V Leighl N Mezger J Archer V Moore N Manegold C Overall survival with cisplatin-gemcitabine and bevacizumab or placebo as first-line therapy for nonsquamous non-small-cell lung cancer: results from a randomised phase III trial (AVAiL) Ann Oncol 2010 21 1804 1809 10.1093/annonc/mdq020 20150572 PMC2924992

